# Remarkably enhanced current-driven 360° domain wall motion in nanostripe by tuning in-plane biaxial anisotropy

**DOI:** 10.1038/s41598-017-13657-w

**Published:** 2017-10-17

**Authors:** Yuanchang Su, Lianghao Weng, Wenjun Dong, Bin Xi, Rui Xiong, Jingguo Hu

**Affiliations:** 1 College of Physics Science and Technology, Yangzhou University, Yangzhou, 225002 People’s Republic of China; 2 School of Physics and Technology, Wuhan University, Wuhan, 430072 People’s Republic of China

## Abstract

By micromagnetic simulations, we study the current-driven 360° domain wall (360DW) motion in ferromagnetic nanostripe with an in-plane biaxial anisotropy. We observe the critical annihilation current of 360° domain wall can be enhanced through such a type of anisotropy, the reason of which is the suppression of out-of-plane magnetic moments generated simultaneously with domain-wall motion. In details, We have found that the domain-wall width is only related to *K*_*y*_ − *K*_*x*_, with *K*_*x*(*y*)_ the anisotropy constant in *x*(*y*) direction. Taking domain-wall width into consideration, a prior choice is to keep *K*_*y*_ ≈ *K*_*x*_ with large enough *K*. The mode of domain-wall motion has been investigated as well. The traveling-wave-motion region increases with *K*, while the average DW velocity is almost unchanged. Another noteworthy feature is that a Walker-breakdown-like motion exists before annihilation. In this region, though domain wall moves with an oscillating behavior, the average velocity does not reduce dramatically, but even rise again for a large *K*.

## Introduction

Current-induced domain wall (DW) motion, which has potential application to the next generation data storage^1^ and logic devices^2^, has attracted much interest in recent years. The motion of transverse domain wall (TDW) due to a spin transfer torque of electrons has been extensively studied^3–11^. For instance, it is well known that the velocity of TDW increases linearly with the applied current. However, for a driven current larger than a critical value, TDW motion would suffer from Walker breakdown^12–14^. Recently, 360° domain wall (360DW), which is formed by the combination of two TDWs with opposite orientations, has attracted much attention^15–33^. One of the advantages is that 360DW shows much weaker stray field as compared to TDW, allowing more stable packing density^27^. The field- and current-driven behaviors of 360DW also differ qualitatively from those of TDW^18^. For example, 360DW may move along a magnetic stripe like TDW under a small driven current, but it will annihilate when the driven current is above a critical value *u*_*c*_^18,26,33^. These characteristics may lead to interesting applications to DW devices^16,18,20,24,25^, and may be also more suitable for building racetrack memory^27,28,30,32^. Unfortunately, the annihilation current for 360DW is significantly small, even smaller than the Walker breakdown current of its constituent part, i.e., a TDW. Even though the annihilation may give rise to the application of spin-wave generators^18^, it may be unfavorable to other application (such as racetrack memory) where fast domain-wall motion is demanded. This poses a challenge to enhance the stability of 360DW under large current, i.e., to raise the critical current *u*_*c*_ for the annihilation.

Recently, there are intriguing experimental progresses on biaxial anisotropy materials^34,35^. It is known such a magnetic anisotropy plays a crucial role in domain-wall formation as well as propagation. One may also expect this kind of anisotropy may be useful for 360DW case. On one side, 360DW is created from two transverse 180DWs, which have opposite topological charges. It is then stabilized through a balance between exchange and demagnetization energies, however, the stability of which may be broken by increasing of out-of-plane magnetic moment generated by fast domain-wall motion, where attractive demagnetization energies reduce accordingly^26,33^. On the other side, supposing magnetic moments are constrained in *xy* plane, then the degree of spin freedom is reduced to 1. Thus 360DW corresponds to a nonsingular topological object, i.e., kink in the 1d xy model^36,37^. However, this topological protection would be broken without such a constraint, since *z*–component of magnetic moments easily lowers the energy of a kink, or equivalently, there is no topological object in 1d classical Heisenberg model^36,37^. Therefore, a suppression of out-of-plane magnetic moment by means of in-plane magnetic anisotropy may be feasible and necessary.

**Figure 1:**
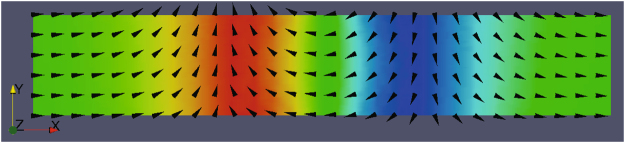
Schematic illustration of the magnetic nanostripe. *x*,*y* and *z* directions are listed in the left corner. A 360DW is initially placed in the center of the stripe. For simplicity, only domain-wall part is presented.

In this paper, we study the effects of a biaxial magnetic anisotropy on current-driven behavior of 360DW in a magnetic nanostripe by micromagnetic simulations. For simplicity, we consider a situation where two easy axes locate at *x* and *y* directions, respectively (see Fig. 1 for more details). We have found that the domain-wall width is only related to *K*_*y*_ − *K*_*x*_. In principle, for a given *K*_*x*_, *u*_*c*_ would always increase with *K*_*y*_. However, for *K*_*y*_ > *K*_*x*_, domain-wall width is broadening considerably. Thus, a prior solution is to keep *K*_*y*_ ≈ *K*_*x*_ with a possibly largest *K*. We also observe two types of domain-wall motion before annihilation. In the range of small current, the average velocity of 360DW increases linearly with the current (traveling-wave motion) and is almost independent of the in-plane biaxial anisotropy. On the other side, in the range of large current, the displacement, time-dependent velocity and the out-of-plane magnetic moment of 360DW oscillate synchronously, exhibiting a Walker-breakdown-like behavior, while the structure of 360DW keeps stable. The average velocity of 360DW does not reduce dramatically, or even re-rise for a large enough *K*.

## Model

As shown in Fig. 1, the magnetic nanostripe (Permalloy stripe) used in this paper is 4096 *nm* long in the *x* direction, 48 *nm* wide in the *y* direction and 5 *nm* thick in the *z* direction. For a thin enough film, the magnetization in the *z* direction should be uniform. Thus we can treat such a system as a two-dimensional one. In the initial state, a 360DW is placed in the center of the nanostripe. In such a magnetic nanostripe, there are sizeable distortions occurring in left-end and right-end edges of the stripe, which may somewhat affect the dynamics of the DWs. In order to avoid the distortions, the local magnetization in the left-end and right-end edges of the stripe are pinned in plane. The Hamiltonian of the stripe with magnetic anisotropy can be written as

1 $$H=-{J}_{ex}\,\sum _{\langle i,j\rangle }\,{{\bf{M}}}_{i}\cdot {{\bf{M}}}_{j}-\sum _{i}\,{K}_{\alpha }{({M}_{i}^{\alpha })}^{2}+\frac{\omega }{2}\,\sum _{i\ne j}\,[\frac{{{\bf{M}}}_{i}\cdot {{\bf{M}}}_{j}}{{r}_{ij}^{3}}-3\frac{({{\bf{r}}}_{ij}\cdot {{\bf{M}}}_{i})\,({{\bf{r}}}_{ij}\cdot {{\bf{M}}}_{j})}{{r}_{ij}^{5}}]$$

where **M**_*i*_ and **M**_*j*_ are the unit magnetization vector for cells *i* and *j*. *J*_*ex*_ is the exchange coupling constant for nearest neighbor cells. *K*_*α*_ (>0) is the anisotropic constant, and *α* can be *x*, *y* or *z*. The third term is the long-range dipole-dipole coupling, where **r**_*ij*_ denotes the lattice vector between cells *i* and *j*. *ω* is the dipole-dipole coupling parameter. The saturation magnetization and the exchange stiffness used in this paper are *M*_*s*_ = 8.6 × 10^5^ *A*/*m* and *A* = 1.3 × 10^−11^ *J*/*m*, respectively. Parameters *J*_*ex*_ and *ω* can be obtained by *J*_*ex*_ = *Aa* and $\omega =\frac{{\mu }_{0}}{4\pi }{M}_{S}^{2}{V}_{cell}$ ^38,39^, where *V*_*cell*_ = *a* ^2^ *b*.

## Result

### Biaxial anisotropy case with *K*_*x*_ = *K*_*y*_

**Figure 2:**
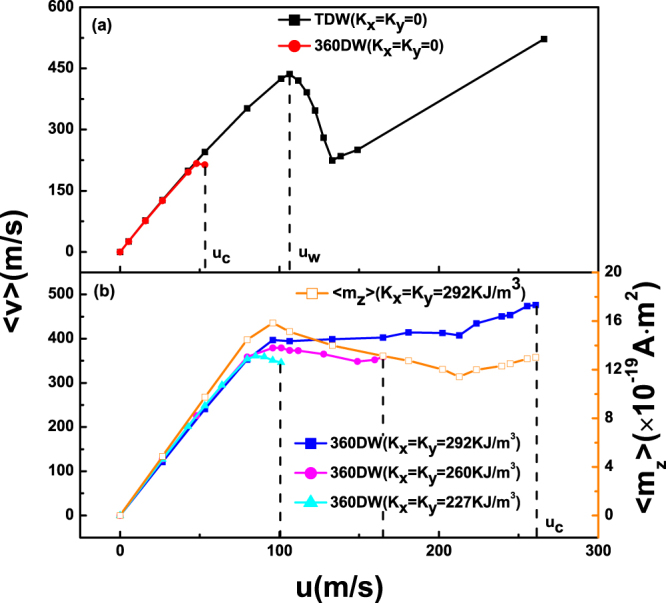
Average velocity 〈*v*〉 of TDW and 360DW as a function of spin current velocity *u* with different in-plane biaxial anisotropy (solid symbols), where *K*_*x*_ = *K*_*y*_ and *K*_*z*_ = 0 have been set. The doted lines and dashed line denote the critical current *u*_*c*_ for the annihilation of 360DW and the Walker breakdown current *u*_*w*_ of TDW, respectively. *m*_*z*_ is the *z*-axis component of total magnetic moment of the 360DW, which is calculated within the area of 360DW (ranges about 120 *nm* in *x* and 48 *nm* in *y*). The average magnetization 〈*m*_*z*_〉 for *K*_*x*_ = *K*_*y*_ = 292 *KJ*/*m* ^3^ is plot with open orange squares.

Based on a sequence of calculations, we find an effective way to enhance *u*_*c*_ by taking both easy x-axis and y-axis anisotropy into account. Figure 2 shows average velocity 〈*v*〉 (defined by a convergent value of displacement over time) of 360DW as a function of spin current velocity *u* with different values of biaxial anisotropy, where *K*_*x*_ = *K*_*y*_ and *K*_*z*_ = 0 have been set. As a benchmark, we plot *u* − 〈*v*〉 curve of TDW with *K*_*x*_ = *K*_*y*_ = *K*_*z*_ = 0 in Fig. 2(a) as well (It should be noticed that, anisotropy can also enlarge the Walker breakdown current *u*_*w*_ of TDW^40,41^). One could immediately see that, without any uniaxial anisotropy, the 〈*v*〉 of 360DW soon annihilates thoroughly after a short linear increasing region. We then define the annihilation point as the critical current *u*_*c*_. Here, a vortex core emerges first and soon move out of the nanostripe. Thus the annihilation is irreversible. As a contrast, an antivortex appears in TDW case, which induces an oscillation in both the magnetic configuration and the velocity^18^. As *u* further increases, TDW would turn into a vortex wall, and one can observes the rapid increment of 〈*v*〉 for the vortex wall. Another distinction is *u*_*c*_ is significantly smaller than the benchmark *u*_*w*_, which has also been reported before^18,26,33^.

In Fig. 2(b), we take *K*_*x*_ = *K*_*y*_ > 0 into account, one can see both *u*_*c*_ and linear increasing region indeed increase with *K*_*x*,(*y*)_, and can easily go beyond *u*_*w*_. In addition, one can notice there are two or three regions of domain-wall motion before it annihilates, depending on the strength of anisotropy. Taking the curve *K*_*x*_ = *K*_*y*_ = 292 *KJ*/*m* ^3^ as an example, in the first region, i.e., *u* < 81 *m*/*s*, the average velocity of 360DW just increases linearly with the current. In fact, comparing to the other values of *K*_*x*,(*y*)_, the linear slope is almost unchanged. One then can deduce that biaxial anisotropy does not need any expense of DW velocity. When *u* is larger than 81 *m*/*s*, an oscillating region emerges, and 〈*v*〉 sightly drops. At last, only in the case of large anisotropy, as in this example, we observe an re-ascendance region just before annihilation.

It is known that domain-wall velocity depends on both *u* and *m*_*z*_, where *m*_*z*_ denotes the *z*-axis component of total magnetic moment of 360DW, which is calculated within the area of 360DW (ranges about 120 in *x* and 48 *nm* in *y*). Since *u* simply increases here, we plot the corresponding average magnetization in *z* direction 〈*m*_*z*_〉 (defined by an average value of *m*_*z*_ in the same time interval) to explore the possible reason behind this unique behavior. As displayed by the open orange square, one can see: 1) 〈*m*_*z*_〉 simply increases in the first linearly increasing region; 2) in the sightly dropped region, the decline of 〈*m*_*z*_〉 compete with the ascent of *u*; 3) in the last region, 〈*m*_*z*_〉 increases again and leads to the second rise of 〈*v*〉.

**Figure 3:**
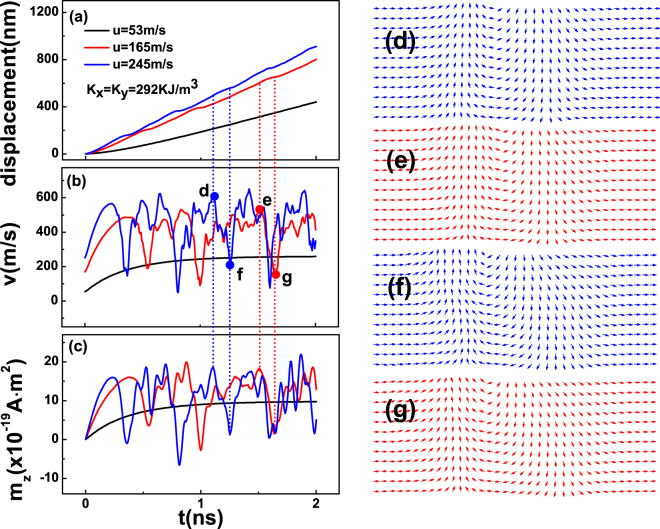
(**a**) Displacement, (**b**) time-dependent velocity *v* and (**c**) the z-axis component of the magnetic moment *m*_*z*_ of 360DW as a function of time under different currents. The biaxial anisotropy is set as *K*_*x*_ = *K*_*y*_ = 292 *KJ*/*m* ^3^. (**d**,**e**) The domain-wall snapshots at the peaks for *u* = 245 *m*/*s* and *u* = 165 *m*/*s*, respectively. (**f**,**g**) The domain-wall snapshots at the valleys for *u* = 245 *m*/*s* and *u* = 165 *m*/*s*, respectively.

To explore the characteristic of domain-wall motion further, we present the details of these three types of domain-wall motion in Fig. 3, adopting *u* = 53 *m*/*s*, 165 *m*/*s* and 245 *m*/*s* with *K*_*x*_ = *K*_*y*_ = 292 *KJ*/*m* ^3^ as three typical examples. In Fig. 3(a), we show the displacement of 360DW. In the case of *u* = 53 *m*/*s*, 360DW exhibits a stationary behavior and moves rigidly with a stationary velocity. Whereas for *u* = 165 *m*/*s* and 245 *m*/*s*, 360DW moves with a oscillation behavior. For a better sense of 360DW motion, we further present instant time-dependent velocity *v* and the z-axis component of the magnetic moment *m*_*z*_ of 360DW as a function of time in Fig. 3(b,c), respectively. One can notice, for *u* = 53 *m*/*s*, both *v* and *m*_*z*_ soon become constant. As a contrast, for the cases of *u* = 165 *m*/*s* and 245 *m*/*s*, *v* and *m*_*z*_ show quasi-periodical oscillations, and the position of peaks and valleys match exactly to the displacement. The oscillation results in the poor linearity of 〈*v*〉 − *u* characteristic in the range of *u* > 81 *m*/*s* (shown in Fig. 2(b)), presenting a Walker-breakdown behavior. Interestingly, the average velocity of 360DW in large current does not reduce dramatically.

We also plot the domain-wall snapshots at the peaks and valleys. As shown in Fig. 3(d–g), one can see the 360DW expands and contracts quasi-periodically, however, keeps stable. When 360DW contracts, both *m*_*z*_ and velocity increase. On the other hand, the expansion would oppose motion. As a result, one could observe a Walker-breakdown-like behavior. This mechanism is different to the stalled anti-vortex nucleation^42^ or a complete suppression of the anti-vortex^14^ in the TDW case.

### Various combination of anisotropy

In order to further understand the effective enhancement of *u*_*c*_ caused by the biaxial anisotropy with *K*_*x*_ = *K*_*y*_, we then investigate the effects of various combination of biaxial anisotropy on *u*_*c*_ for a comparison. We first start with the simplest case, i.e., uniaxial anisotropy.

**Figure 4:**
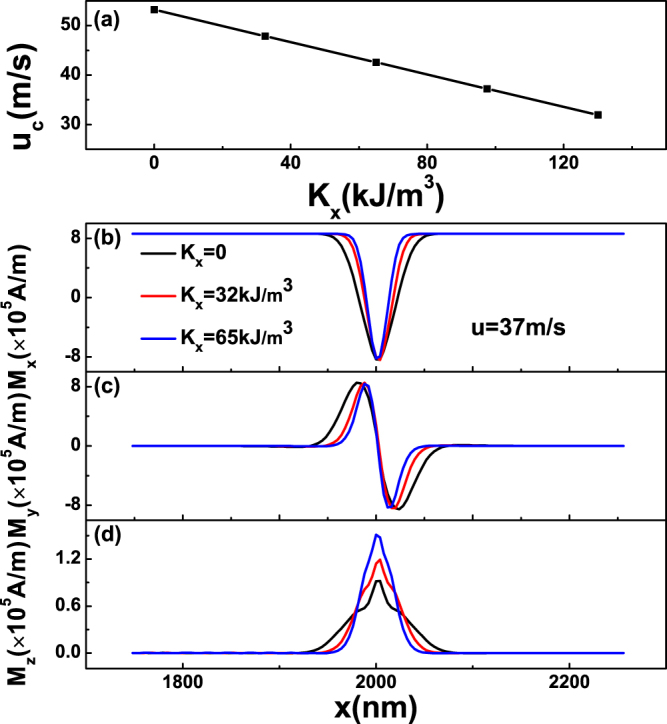
(**a**) Critical current *u*_*c*_ for the annihilation of 360DW as a function of *K*_*x*_, where *K*_*z*_ = 0 and *K*_*y*_ = 0 have been set. (**b**) The *x*-axis component *M*_*x*_, (**c**) The *y*-axis component *M*_*y*_ and (**d**) The *z*-axis component *M*_*z*_ of the local magnetization on *y* = 24 *nm* as a function of *x* with different *K*_*x*_, where small current *u* = 37 *m*/*s* is applied.

In Fig. 4, we present the results with only *x*–direction anisotropy. One can see that in subpanel (a), *u*_*c*_ decreases with the increasing of *K*_*x*_, and subpanels (b)-(d) show the local magnetization in *x*, *y* and *z* directions, respectively. From all plots of magnetization, one can see that domain-wall width shrinks (we define the domain-wall width as the region of *M*_*z*_ ≠ 0 or *M*_*x*_ ≠ *M*_*s*_). However, the maximum of *M*_*x*_ and *M*_*y*_ almost not change, while the peak of *M*_*z*_ increases. It suggests that the width of 360DW as well as *M*_*z*_ plays an important role to *u*_*c*_. In the case of increasing *K*_*x*_, the width of 360DW should decrease. Then, the local magnetization vector near and at the area of 360DW should turn out of plane to reduce the exchange coupling energy. As a result, the *M*_*z*_ of the 360DW increases, which deduces the contribution of demagnetization energy, leading to the decreasing of *u*_*c*_.

**Figure 5:**
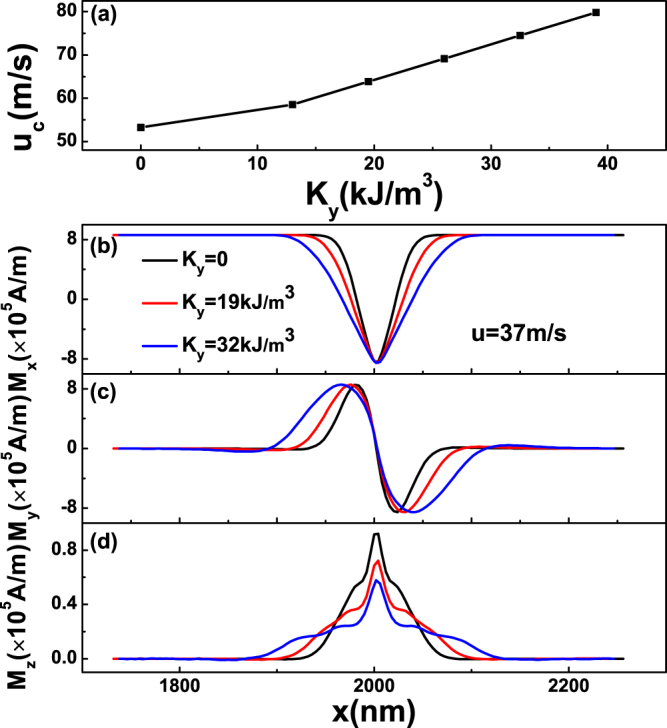
(**a**) Critical current *u*_*c*_ for the annihilation of 360DW as a function of *K*_*y*_, where *K*_*x*_ = 0 and *K*_*z*_ = 0 have been set. (**b**) The *x*-axis component *M*_*x*_, (**c**) The *y*-axis component *M*_*y*_ and (**d**) The *z*-axis component *M*_*z*_ of the local magnetization on *y* = 24 *nm* as a function of *x* with different *K*_*y*_, where small current *u* = 37 *m*/*s* is applied.

We now turn to the case with only *K*_*y*_. As shown in Fig. 5. One can observe that both *u*_*c*_ and domain-wall width increases with the increasing of *K*_*y*_ in Fig. 5(a–d), respectively. As a contrast to the result under only *K*_*x*_ ≠ 0, the width of 360DW now increases with *K*_*y*_. Another characteristic is only the maximum of *M*_*z*_ shown in Fig. 5(d) decreases with *K*_*y*_, which supports the significance of *M*_*z*_. As *K*_*y*_ increasing, the descend of *M*_*z*_ mainly comes from the in-plane anisotropic field. The increasing of *u*_*c*_ can be owed to the decreasing of the *M*_*z*_ of 360DW, preserving the demagnetization energy. Even though the *u*_*c*_ can be enhanced by tuning *K*_*y*_, it is not a good choice because the width of 360DW is significantly enhanced, which is not suitable for practical use.

**Figure 6:**
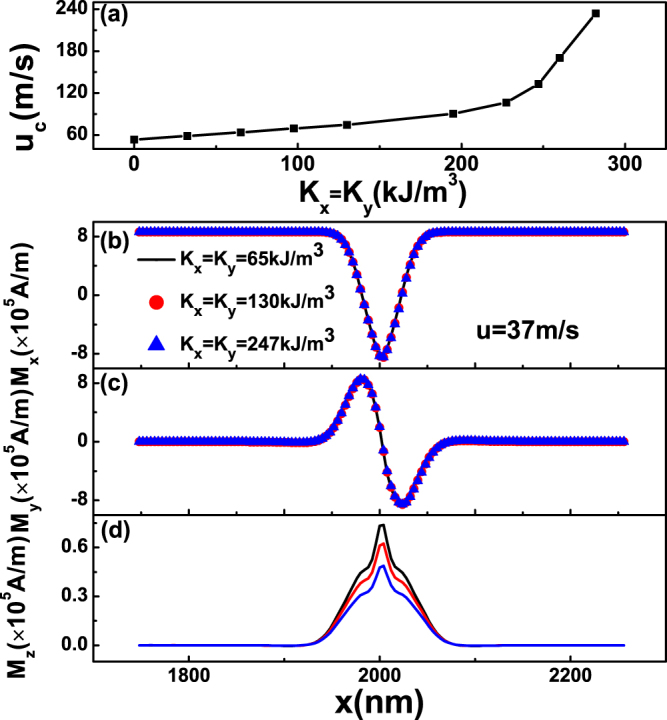
(**a**) Critical current *u*_*c*_ for the annihilation of 360DW as a function of *K*_*x*_ = *K*_*y*_, where *K*_*z*_ = 0 have been set. (**b**) The *x*-axis component *M*_*x*_, (**c**) The *y*-axis component *M*_*y*_ and (**d**) The *z*-axis component *M*_*z*_ of the local magnetization on *y* = 24 *nm* as a function of *x* with different *K*_*x*_ = *K*_*y*_, where small current *u* = 37 *m*/*s* is applied.

Based on the results shown in Figs 4 and 5, one can naturally realize that *u*_*c*_ may be effectively enhanced simultaneously with a fixed DW width by the combination of *K*_*x*_ and *K*_*y*_. As shown in Fig. 6(a–d), *u*_*c*_ indeed increases with equal biaxial anisotropy, i.e., *K*_*x*_ = *K*_*y*_, and the width of 360DW almost remains unchanged due to the different effects of *K*_*x*_ and *K*_*y*_ on DW width. The increasing of *u*_*c*_ is owed to the decreasing of the *M*_*z*_ of 360DW, which is demonstrated clearly in Fig. 6(d).

**Figure 7:**
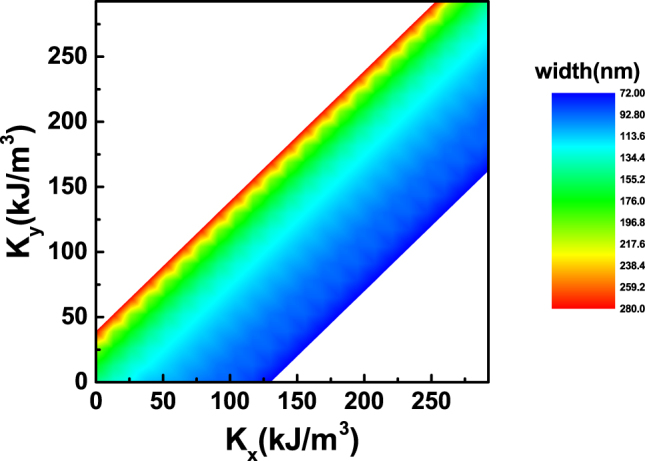
Contour plot of domain-wall width against *K*_*x*_ and *K*_*y*_.

In the Fig. 7, we plot the contour plot of domain-wall width against *K*_*x*_ and *K*_*y*_. It can be clearly seen from the plot that the domain-wall width only depends on the value of *K*_*y*_ − *K*_*x*_. As for *K*_*x*_ > *K*_*y*_, i.e., the lower right part of Fig. 7, domain-wall width increases slowly with the value of *K*_*y*_ − *K*_*x*_. One can see that from *K*_*x*_ − *K*_*y*_ = 125 *KJ*/*m* ^3^ to *K*_*x*_ = *K*_*y*_, the width only changes from 72 to 155. However, as for *K*_*y*_ > *K*_*x*_, i.e., the upper left part of Fig. 7, the width increases rapidly from 155 *nm* to 280 *nm*, corresponding *K*_*y*_ = *K*_*x*_ to *K*_*y*_ − *K*_*x*_ = 40 *KJ*/*m* ^3^. Besides these regions, 360DW becomes unstable in our simulations, which corresponds to the white area in the contour plot.

**Figure 8:**
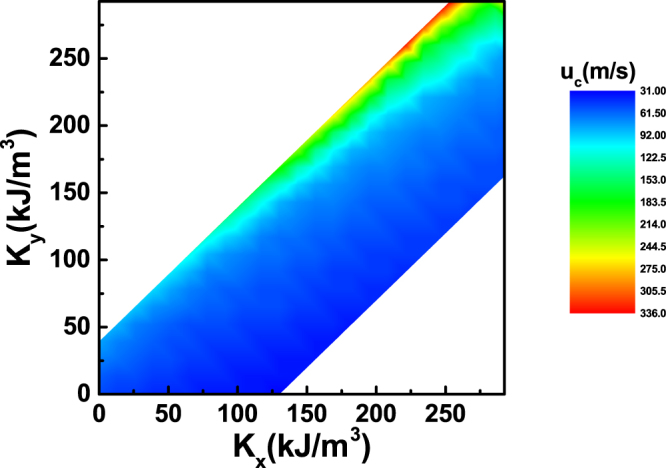
Contour plot of critical current *u*_*c*_ against *K*_*x*_ and *K*_*y*_.

We finally plot the contour plot of *u*_*c*_ against *K*_*x*_ and *K*_*y*_ in the Fig. 8. From the contour plot, one can learn that in principle, *u*_*c*_ would always increase with *K*_*y*_ for a fixed *K*_*x*_. However, for *K*_*y*_ > *K*_*x*_, domain-wall width is broadening considerably. Thus, a prior solution is to keep *K*_*y*_ ≈ *K*_*x*_ with a possibly largest *K*_*x*_.

## Discussion

Though previous work mainly focuses on the theoretical simulation, we notice that materials with a biaxial anisotropy have already been realized experimentally, examples ranges from CoFe_2_O_4_ films on (100) MgO substrates^34^ to Fe films on BaTiO_3_ substrates^35^. 360DW on those materials as well as related applications may need further study.

## Conclusions

We have found the motion of 360DW can be well promoted by introducing an biaxial anisotropy. In general, the critical annihilation current of 360DW would always increase with *K*_*y*_. However, taking domain-wall width into consideration, we found the case of *K*_*x*_ ≈ *K*_*y*_ would be a better choice. In this case, the width of 360DW almost remains unchanged due to the different effects of *K*_*x*_ and *K*_*y*_ on DW width, meanwhile, out-of-plane magnetic moments are suppressed. We also observe two types of DW motion. For the range of small current, both the linearity and slope of velocity-current characteristics remains unchanged, i.e., independent to the anisotropy. On the other side, for large current, though a Walker-breakdown-like behavior appears, the average velocity of 360DW does not reduce dramatically. We hope our findings could shed lights on the potential applications of 360DW.

## Method

In simulations, the unit cell size (*a* × *a* × *b*) is 4 *nm* × 4 *nm* × 5 *nm*. In order to minimize the edge effect of demagnetization energy in the moving direction, a scheme that keeps the 360DW centered in the stripe has been used. We use the Landau-lifshitz-Gilbert (LLG) equation with additional adiabatic and nonadiabatic spin-transfer torque^43,44^ to describe the magnetization dynamics. When the current is applied along +*x* direction, the LLG equation is

2 $$\frac{\partial {\bf{M}}}{\partial t}=-\gamma {\bf{M}}\times {{\bf{H}}}_{eff}+\alpha {\bf{M}}\times \frac{\partial {\bf{M}}}{\partial t}+u{\bf{M}}\times ({\bf{M}}\times \frac{\partial {\bf{M}}}{\partial x})+\beta u{\bf{M}}\times \frac{\partial {\bf{M}}}{\partial x},$$

where ${{\bf{H}}}_{eff}=-\frac{\partial H}{\partial {\bf{M}}}$ is the effective field acting on the unit magnetization vector **M**, *γ* is the gyromagnetic ratio, *α* is the Gilbert damping constant, *β* denotes the non-adiabatic spin torque coefficient, *u* is the spin current velocity defined as *u* = *jPgμ*_*B*_/2*eM*_*s*_ with *P* the spin polarization, *j* the current density, *μ*_*B*_ the Bohr magneton and *e* the electron charge, respectively. In this paper, we set *α* = 0.01 and *β* = 0.05 according to previous works^18,44^.
